# The Impact of Uremic Toxicity Induced Inflammatory Response on the Cardiovascular Burden in Chronic Kidney Disease

**DOI:** 10.3390/toxins10100384

**Published:** 2018-09-23

**Authors:** Ligia Maria Claro, Andrea N. Moreno-Amaral, Ana Carolina Gadotti, Carla J. Dolenga, Lia S. Nakao, Marina L.V. Azevedo, Lucia de Noronha, Marcia Olandoski, Thyago P. de Moraes, Andréa E. M. Stinghen, Roberto Pécoits-Filho

**Affiliations:** 1 Graduate Program in Health Sciences, School of Medicine, Pontifícia Universidade Católica do Paraná, Curitiba, PR 80215-901, Brazil; lmclaro@gmail.com (L.M.C.); deamoreno@gmail.com (A.N.M.-A.); ana_raixu3@hotmail.com (A.C.G.); malu_tu@yahoo.com.br (M.L.V.A.); lnno.noronha@gmail.com (L.d.N.); bio.estatistica@pucpr.br (M.O.); thyago.moraes@pucpr.br (T.P.d.M.); 2 Basic Pathology Department, Universidade Federal do Paraná, Curitiba, PR 80050-540, Brazil; carla.vet10@gmail.com (C.J.D.); lia.nakao@ufpr.br (L.S.N.); andreastinghen@ufpr.br (A.E.M.S.)

**Keywords:** uremic toxins, inflammatory biomarkers, sCD36, sRAGE, fractalkine (CX3CL1), fractalkine receptor (CX3CR1)

## Abstract

Uremic toxin (UT) retention in chronic kidney disease (CKD) affects biological systems. We aimed to identify the associations between UT, inflammatory biomarkers and biomarkers of the uremic cardiovascular response (BUCVR) and their impact on cardiovascular status as well as their roles as predictors of outcome in CKD patients. CKD patients stages 3, 4 and 5 (*n* = 67) were recruited and UT (indoxyl sulfate/IS, p-cresil sulfate/pCS and indole-3-acetic acid/IAA); inflammatory biomarkers [Interleukin-6 (IL-6), high sensitivity C reactive protein (hsCRP), monocyte chemoattractant protein-1 (MCP-1), soluble vascular adhesion molecule-1 (sVCAM-1), soluble intercellular adhesion molecule-1 (sICAM-1) and soluble Fas (sFas)] and BUCVRs [soluble CD36 (sCD36), soluble receptor for advanced glycation end products (sRAGE), fractalkine] was measured. Patients were followed for 5.2 years and all causes of death was used as the primary outcome. Artery segments collected at the moment of transplantation were used for the immunohistochemistry analysis in a separate cohort. Estimated glomerular filtration rate (eGFR), circulating UT, plasma biomarkers of systemic and vascular inflammation and BUCVR were strongly interrelated. Patients with plaque presented higher signs of UT-induced inflammation and arteries from CKD patients presented higher fractalkine receptor (CX3CR1) tissue expression. Circulating IS (*p* = 0.03), pCS (*p* = 0.007), IL-6 (*p* = 0.026), sFas (*p* = 0.001), sCD36 (*p* = 0.01) and fractalkine (*p* = 0.02) were independent predictors of total mortality risk in CKD patients. Our results reinforce the important role of uremic toxicity in the pathogenesis of cardiovascular disease (CVD) in CKD patients through an inflammatory pathway.

## 1. Introduction

Cardiovascular disease (CVD) is the main cause of morbidity and mortality of chronic kidney disease (CKD) patients [1,2] and the high CVD risk cannot be fully explained by traditional risk factors (hypertension, diabetes, dyslipidemia) [2]. CKD specific risk factors have been described as ‘missing links’ that explain the high cardiovascular burden in CKD [3,4]. Uremic toxicity has been highlighted as a pivotal risk factor for CVD in CKD. 

Uremic toxins (UTs) cause cell or system dysfunction, and experimental studies have described that the immune activation induced by several UTs [5,6,7,8] is associated with circulating UT levels and inflammatory biomarkers [5,8,9]. In order for us to understand the mechanisms relating the immuno-inflammatory response to uremic toxicity and its CVD consequences, we need a comprehensive approach to the UT analysis, circulating biomarkers of systemic and vascular inflammation, and particularly inflammatory mediators that capture the specific immune response to uremic toxicity. 

We hypothesize that circulating UT [indoxyl sulfate (IS), p-cresyl sulfate (p-CS) and indole-3-acetic acid (IAA)] are associated with inflammatory [Interleukin-6 (IL-6), high sensitivity C-reactive protein (hsCRP)], and vascular inflammation [monocyte chemoattractant protein-1 (MCP-1), soluble vascular adhesion molecule-1 (sVCAM-1), soluble intercellular adhesion molecule-1 (sICAM-1) and soluble Fas (sFas)] biomarkers, including those particularly involved in mechanisms related to the cardiovascular response to specific uremic toxins [soluble CD36 (sCD36), soluble receptor for advanced glycation end products (sRAGE) and fractalkine]. These biomarkers were previously described to be activated specifically by the UTs, oxidized LDL, advanced glycation end products (AGEs) and indoxil sulphate, respectively. In this study, these biomarkers will be named as biomarkers of the uremic cardiovascular response-BUCVR. In addition, we hypothesize that UT-induced inflammatory reaction leads to vascular structural and histological changes and to increased mortality risk. To advance in the understanding of mechanisms linking UT to cardiovascular (CV) burden in CKD, we aim to analyses the relationship between UTs, inflammatory biomarkers and BUCVRs and their association with atherosclerotic CVD and mortality risk in patients with moderate to severe CKD. 

## 2. Results

The demographic, clinical and laboratory characteristics of chronic kidney disease (CKD) population of the study are shown in Table 1.

Patients were divided according to the stages of CKD (1 to 5) and, most were in stage 3 (31%) and stage 4 (22%). Patients were taking angiotensin-converting enzyme inhibitors in 69% of the cases, diuretics in 57%, aspirin in 54% and statins in 13% of cases. No patients were in treatment with erythropoietin stimulants or vitamin D analogues.

The demographic, clinical and laboratory characteristics of CKD transplant recipients are shown in Table 2.

Most patients (86%) were treated with hemodialysis before the transplantation and remaining were on peritoneal dialysis. The mean time on renal replacement therapy was 37 ± 8 months.

The overall results for UT, inflammatory biomarkers and BUCVR in the study population are described on Table 3.

### 2.1. Correlations between eGFR and Uremic Toxins, Inflammatory Biomarkers and BUCVR 

**Figure 1:**
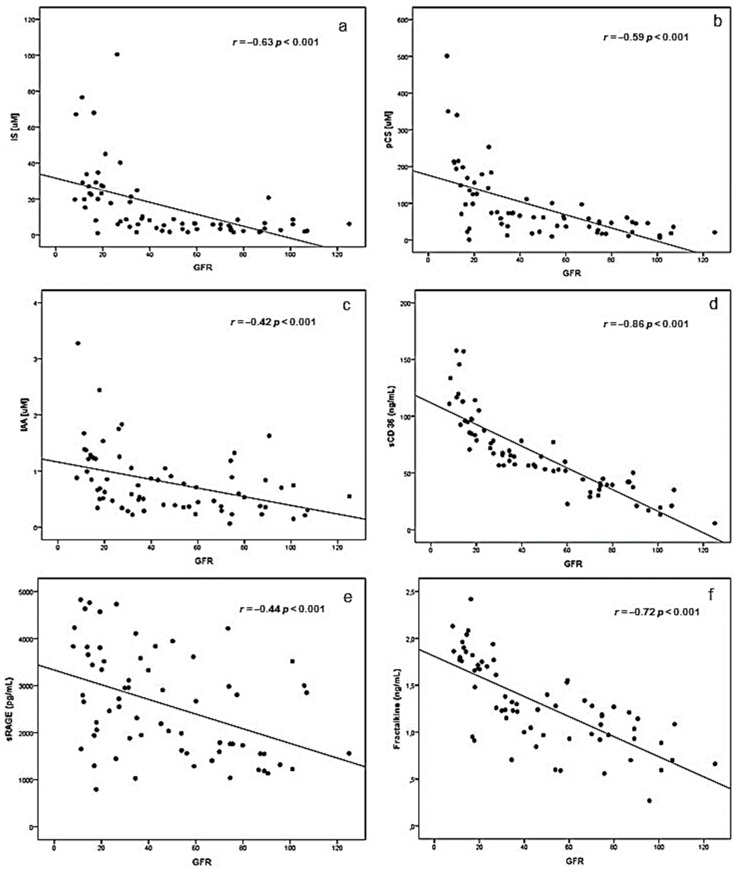
The scatter plots (**a**–**c**) show the relationship between the concentrations of UTs and glomerular filtration rate (GFR) in CKD patients in pre-dialysis. Similarly, the scatter plots (**d**–**f**) show the correlation with biomarkers of the uremic cardiovascular response and GFR.

We observed a negative correlation between eGFR and UTs (IS, pCS and IAA) (Figure 1a–c). No significant correlations were observed between GFR and IL-6 and hsCRP. In contrast, we observed negative correlations between eGFR and biomarkers of vascular inflammation [MCP-1 (*r* = −0.28, *p* < 0.05), sVCAM-1 (*r* = −0.35, *p* < 0.005), sFas (*r* = −0.49, *p* < 0.001)] but not to sICAM-1. In addition, we observed strong correlations between eGFR and BUCVRs (sCD36 (*r* = −0.86, *p* < 0.001), sRAGE (*r* = −0.44, *p* < 0.001) and fractalkine (*r* = −0.72, *p* < 0.001) (Figure 1d–f).

### 2.2. Correlations between Plasma Levels Uremic Toxins, Inflammatory Biomarkers and BUCVR 

In the analysis of inflammatory biomarkers, MCP-1 correlated positively with IS, pCS and IAA (*r* = 0.38; *r* = 0.42; *r* = 0.29; *p* = 0.001, *p* < 0.001 and *p* = 0.015, respectively). sVCAM-1 showed correlation with IS, pCS and IAA (*r* = 0.29; *r* = 0.29; *r* = 0.27; *p* = 0.019, 0.017 and 0.025 respectively). IL-6, hsCRP and sICAM-1 were not correlated with any of the UTs. sFas showed positive correlations with IS (*r* = 0.39; *p* = 0.001) and pCS (*r* = 0.46; *p* < 0.001) but no significant correlations with IAA (Table 4).

sCD36 presented a moderate correlation with IS, pCS and IAA (*r* = 0.62; *r* = 0.55, *r* = 0.52; *p* < 0.001 respectively). Fractalkine had a strongly positive correlation with IS and pCS (*r* = 0.77; *p* < 0.001 for both). There was no significant correlation between IAA and biomarker sRAGE (*r* = 0.20; *p* = 0.108). For other BUCVRs, the IAA showed a weak correlation (Table 5)

There was no significant difference between plasma levels of inflammatory biomarkers (hsCRP, MCP-1, sVCAM-1 and sICAM-1) and BUCVR in patients with or without the following comorbidities: diabetes, obesity, hypertension, dyslipidemia, history of CVD and smoking. On the other hand, we observed a significant difference between IL-6 plasma levels and CVD (*p* = 0.036). We also found no differences between race and gender.

Table 6 shows correlations between plasma levels of inflammatory biomarkers and BUCVR. We also observed that MCP-1 had a positive correlation with IL-6 (*r* = 0.36, *p* = 0.003), sVCAM-1 (*r* = 0.41, *p* = 0.001) and all BUCVRs while, sICAM-1 correlated only with hsCRP (*r* = 0.37, *p* = 0.002). sFas correlated positively with all BUCVRs but not with the inflammation markers. With respect to BUCVR, we observed that only sCD36 correlated positively with sVCAM-1. BUCVRs had a significant correlation between each other; however, the relation was the strongest between sCD36 and Fractalkine (*r* = 0.73, *p* < 0.001).

### 2.3. Tissue Expression of Inflammatory Biomarkers and BUCVR in Human Renal Arteries

**Figure 2:**
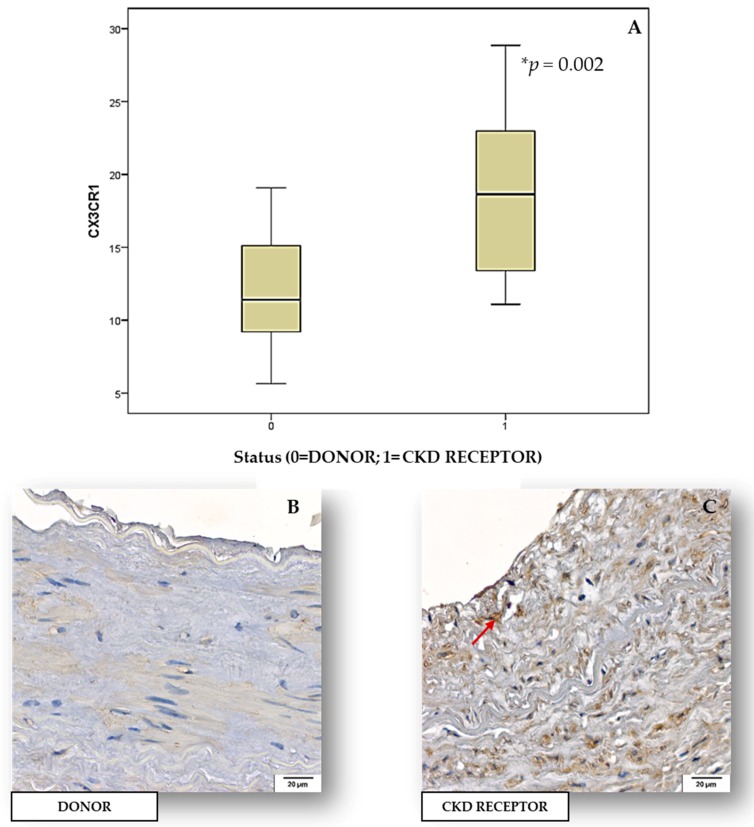
(**A**) Graph with the total percentage (%) of tissue expression of fractalkine receptor (CX3CR1) by immunohistochemistry (IHC). * *p* = 0.002, significant difference between donor and CKD receptor tissue expression of CX3CR1; *t* Student (*p* < 0.05). Demonstrative images showing the negative (**B**) and immunopositivity (**C**) (red arrow) tissue expression of CX3CR1 for medium power field (200×), in the renal arteries.

No statistical difference was observed upon comparing the tissue expression of inflammatory biomarkers (IL-6, MCP-1) and BUCVR (CD36, fractalkine) between human renal arteries of CKD transplant recipients and donor (control). In contrast, we observed that CKD transplant recipient group showed stronger tissue expression for CX3CR1 (*p* = 0.002) when compared to the donor samples (Figure 2).

### 2.4. Comparisons between Plasma Levels Uremic Toxins, Inflammatory Biomarkers, Vascular Inflammation, BUCVR in Patients with and without Carotid Artery Plaques

The *t*-test comparing cases with and without atherogenic plaque relative to UT, inflammatory biomarkers, vascular inflammation and BUCVR showed a statistically significant difference only for sCD36 (*p* = 0.049) (Table 7).

### 2.5. Survival Analysis According to eGFR, Plasma Levels Uremic Toxins, Inflammatory Biomarkers and BUCVR

**Figure 3:**
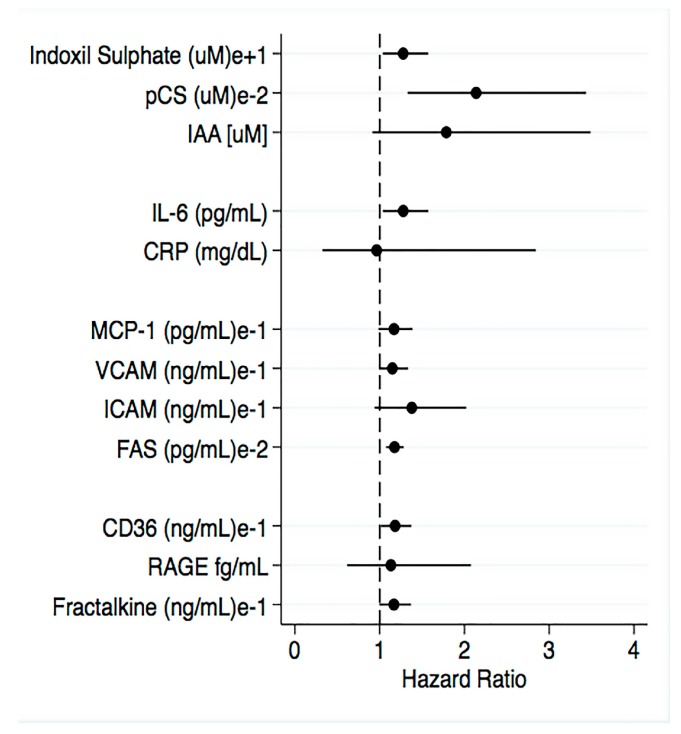
Cox regression univariate analysis for evaluating the relationship between independent co-variables and clinical outcomes in CKD patients of the study.

The survival analysis shows the relationship between independent co-variables and clinical outcomes in CKD patients of the study (Figure 3). After 5.2 years of follow-up, twenty-two patients presented a composite cardiovascular event, which included total cardiovascular mortality, non-fatal myocardial infarction (MI), revascularization, amputation or non-fatal stroke. After follow-up and adjustments for age, sex, diabetes and CVD we observed that lower eGFRs were associated with increased mortality (HR 1.03, IC 95% 1.010–1.060; *p* = 0.02). The highest serum levels of IS (HR 1.02, IC 95% 1.001–1.040; *p* = 0.03), pCS (HR 1.005, IC 95% 1.001–1.010; *p* = 0.007), IL-6 (HR 1.20, IC 95% 1.02–1.40; *p* = 0.026), sFas (HR 1.002, IC 95% 1.003–1.40; *p* = 0.001), sCD36 (HR 1.02, IC 95% 1.004–1.030; *p* = 0.01) and fractalkine (HR 3.98, IC 95% 1.20–13.18; *p* = 0.02) were associated with an increased risk of mortality. On the other hand, hsCPR, MCP-1, sVCAM-1, sICAM-1, sRAGE and IAA were not independent or significant predictors of fatal events in the study population.

## 3. Discussion

The association between general inflammatory markers and poor outcomes in CKD has been consistently described, but the role of inflammatory pathways linking uremic toxicity to CVD is largely unknown [10]. Here we studied a population with moderate to advanced CKD and described (1) strong correlations between eGFR, circulating uremic toxins, serum biomarkers of systemic and vascular inflammation and BUCVR (2) evidence of the impact of inflammation triggered by uremic toxicity in arteries (both in a non-invasive evaluation and also in tissue) of CKD patients; and (3) that several biomarkers of uremic toxicity and inflammation were associated with mortality in patients with moderate to severe CKD.

In agreement with previous studies [9,11,12,13], we observed that UT accumulated in the circulation of CKD patients as kidney function declines. Similarly, MCP-1, sVCAM-1, sFas and several BUCVR are all correlated negatively with eGFR. These correlations have been described in previous isolated studies, however, our study was the first to concomitantly describe the associations between eGFR and UT, inflammatory biomarkers and vascular inflammation, and the first to describe BUCVR in relation to kidney function. We previously observed not only relationships between renal function and various inflammatory biomarkers [14], but also an increased expression of this inflammatory molecules by endothelial cells in vitro [15]. The present results support the hypothesis that uremic toxicity, inflammation and vascular response are interrelated processes. 

In the current study, the correlation between IS, p-CS and IAA levels and the biomarkers of systemic inflammation, namely C-reactive protein (CRP) and IL6 contrasts with a previous study [8] that found that IS and p-CS correlated significantly with IL-6 in stages 3 and 4 CKD patients. Also, Dou et al. [16] found a significant positive correlation between IAA and CRP. On the other hand, IS, p-CS and IAA correlated positively with MCP-1 and sVCAM-1 in the present study. These results corroborate the concept that UT, mainly IS and p-CS, mediate an increased MCP-1 expression, and this could present a possible mechanism for the induction of vascular pro-inflammatory phenotype observed in CKD [5]. Another important observation of the present study is the significant correlation between UT (IS, p-CS) and sFas. This corroborates with the information that sFas has been defined as a marker of inflammation and endothelial dysfunction in adults with coronary artery disease and CKD [17,18]. 

To the best of our knowledge, this is the first study demonstrating a relationship between UT and BUCVR. We observed that plasma levels of two UTs analyzed (IS, p-CS), showed a positive correlation with sCD36, sRAGE and fractalkine. Until now, little is known about the impact of UTs on immune cells and chronic inflammatory responses that can accelerate the development and progression of CVD in CKD patients. A recent study, demonstrated that IS, but not p-CS, induces the secretion of fractalkine by human monocytes, a CX3CR1-specific chemokine ligand, which is highly expressed in lymphocytes [19]. Increased concentrations of sCD36 in CKD patients is another example of the uremia-related immune dysfunction. Previous studies have demonstrated that the ox-LDL, inflammatory chemokines and cytokines induce CD36 expression in CKD [20,21]. 

UTs (mainly IS and p-CS) are implicated in the pathogenesis of atherosclerosis as well as non-atherosclerotic vascular diseases commonly found in the CKD setting [22]. Kashiyama and colleagues (2015) show that plaque volume was significantly greater in CKD 4–5 than in other stages and that CKD stage is an independent predictor of plaque progression [23]. This is explained by the uremic milieu that promotes endothelial dysfunction and increased serum levels of inflammatory markers that play an important role in the development of atherosclerosis [24]. Under these conditions, levels of cell adhesion molecules, such as ICAM-1 and VCAM-1, promote monocyte infiltration into the activated endothelium [25,26]. High levels of CD36 could promote atherogenesis through enhanced ox-LDL clearance and, consequently, through foam cell formation [20]. Higher concentration of CD36 in patients with atherosclerotic plaques supports the hypothesis that this biomarker has pro-atherogenic properties. On other hand, there was no difference between the UTs, fractalkine and sRAGE with presence or absence of plaque. Similarly, in a previous study fractalkine and its receptor (CX3CR1) were increased in circulation as well as in atherosclerotic plaque in coronary artery disease [27], providing a potential link between uremic toxicity and accelerated atherogenesis. Reinforcing this information, through IHC of renal arteries we observed that CKD transplant recipients group showed stronger CX3CR1 tissue expression when compared to the donor samples. Unfortunately, it’s not possible to know precisely which cells types are expressing CX3CR1 in Figure 2 but, the literature shows that CX3CR1 is mainly expressed in T lymphocytes, monocytes, natural killer cells and mast cells [28]. In addition, intimal smooth muscle cells in atherosclerotic vessels and stimulated endothelial cells also express CX3CR1 [29].

In recent meta-analysis, involving patients with CKD stage 3 and above, the authors concluded that elevated levels of p-CS and IS are associated with increased mortality in patients with CKD corroborating with our study that shows an impact of uremic toxicity, inflammation and CKD disease. p-CS, but not IS, is associated with an increased risk of CV events [30]. Dou et al (2015) observed that IAA serum levels were a significant predictor of mortality and cardiovascular events [16]. In disagreement with these findings, our results showed that IAA was not a significant predictor of fatal events in the study population. Similar to protein-bound UTs, higher serum levels of IL-6, sFas, sCD36 and fractalkine were associated with increased risk of mortality. In a different CVD setting, a study assessed fractalkine plasma levels in 349 patients with advanced systolic heart failure. The authors observed over a median follow-up of 4.9 years that fractalkine was a significant predictor of all-cause mortality [31]. Like a fractalkine, sCD36 was also considered a predictor of CV mortality, since sCD36 concentration in dialysis patients were at increased risk of 3-year cardiovascular mortality, as compared to the rest of the cohort [20]. We observed that sRAGE was not a significant predictor of fatal events in the study population, in contrast to previous findings that showed elevated sRAGE levels associated with adverse outcomes in patients with CKD [32,33]. In addition, high levels of circulating sRAGE were associated with worse kidney function and increased risk in CKD progression [34].

There are some limitations of the present study. First, our cohort was relatively small and several patients lost to follow up and data should be confirmed in larger CKD populations with a standardized follow up, particularly powered for the survival analysis. Secondly, we did not measure free concentrations of IS and p-CS. Liabeauf et al. (2010) observed that total p-CS levels and free fraction are higher in advanced stage CKD patients, but only free fraction was associated with mortality in these patients [12]. In addition, it can be speculated that the influence of p-CS and IS concentration on overall mortality among CKD patients is more likely to be a group effect for protein-bound solutes as a whole. Furthermore, evidence of increased risk should be reproduced in multiple groups of CKD patients and in a wide range of clinical settings. Thirdly, we measured sRAGE individually, however, ideally the levels of sRAGE should be considered in conjunction with plasma levels of AGEs. In the literature, the lack of consistent association of AGE/RAGE pathway with CKD and its complications, may reflect difficulties in measuring AGE products. Immunologic techniques such as ELISA have been employed more commonly but are much less specific, capturing molecules other than the target [35].

In conclusion, we described several associations between eGFR, circulating UTs, serum biomarkers of systemic and vascular inflammation and BUCVR. We also observed a modest impact of inflammation potentially triggered by uremic toxicity in arteries (both in a non-invasive evaluation-observed specifically for CD36 as well as in tissue—particularly related to CX3CR1) and its association with mortality (taking into consideration the limitations imposed by the potential lack of power of the survival analysis) in this group of CKD patients. Our results reinforce the important role of uremic toxicity in the pathogenesis of CVD in CKD patients through an inflammatory pathway. 

## 4. Materials and Methods 

The study protocol was approved by the Ethics Committee of Pontifical Catholic University of Paraná (Curitiba, Brazil) under registration numbers CEP/PUCPR 509 (approved on 1 June 2005) and CEP/PUCPR 577 (approved on 6 April 2005), respectively and informed consent was obtained from all patients recruited. Detailed information of these cohorts has been published elsewhere [15,36,37].

### 4.1. Subjects

#### 4.1.1. Pre-Dialysis CKD Patients

**Figure 4:**
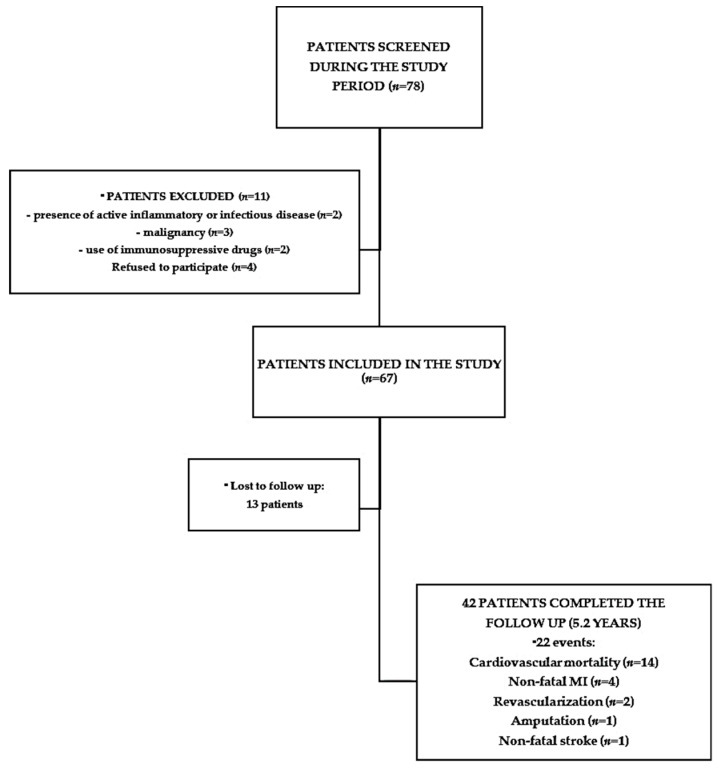
Flow chart of the study.

For the analysis of biomarkers, characterization of CVD and analysis of mortality risk, 67 patients from our CKD clinic were included (cohort 1). Inclusion criteria were age >18 years old, presence of CKD (proteinuria or decreased glomerular filtration rate-GFR) in 3 consecutive evaluations) and willingness to participate in the study. Exclusion criteria were active inflammatory or infectious disease, malignancy and use of immunosuppressive drugs (Figure 4). GFR was estimated by CKD-EPI (Chronic Kidney Disease Epidemiology Collaboration) equation [38]. Diabetes was defined according to a medical history of diabetes, use of hypoglycemic agents or compatible levels of fasting glycemia or HbA1C. Hypertension was defined according to a medical history of hypertension or the use of anti-hypertensive agents at the visit. Cardiovascular disease was defined according to a history of a previous stroke, myocardial infarction or amputation. LVH was documented when the patients presented an echocardiogram reporting LVH. Smoking was defined as previous or present history of smoking any number of tobacco product.

#### 4.1.2. CKD Transplant Recipients

For the evaluation of the tissue expression of biomarkers in arteries, 14 stage 5 CKD patients who underwent a living donor renal transplantation (cohort 2) were included. Their kidney donors served as the control group. Exclusion criteria included smoking, diabetes, systemic inflammatory disease and history or symptoms of CVD prior to the transplantation. Both groups underwent CVD screening within 3 months of the study, including serum biomarkers of CV risk, Framingham score, stress echocardiogram, or scintigraphy. Only patients with low risk for CV events after the evaluation were included in the analysis. 

### 4.2. Material 

#### 4.2.1. Blood Sampling

Peripheral blood samples from patients with pre-dialysis CKD were collected in EDTA. After centrifugation (15 mins, 1200× *g*, 18 °C), the obtained plasma was divided into aliquots and stored at −80 °C until analysis of UT or biomarkers. Patients were classified in stages 1–5 according to the KDIGO recommendations [39].

#### 4.2.2. Renal Arteries Samples

During kidney transplantation, external iliac and renal artery segments were collected from CKD patients and controls, respectively, and were immediately fixed in 5 mL of 10% formalin. After an overnight period, specimens were embedded in paraffin.

#### 4.2.3. Clinical and Biochemical Characteristics of the Patients (Cohort 1)

Data regarding anthropometry, age, gender, primary kidney disease, smoking habit, history of dyslipidemia, diabetes mellitus, hypertension and clinically detected cardiovascular disease (coronary heart disease, cerebrovascular disease, peripheral vascular disease and heart failure) were recorded by a detailed analysis of medical records, patient interview and physical examination. Biochemical parameters were measured by colorimetric methods (Cobas Mira Plus; Roche, Montclair, NJ, USA). 

### 4.3. Measurement of Plasma Levels of Uremic Toxins

Total concentrations of UTs pCS, IS and IAA were quantified by HPLC method (High Performance Liquid Chromatography) with fluorescent detection. The ultrafiltered plasma was injected into HPLC system (Shimadzu Prominence, Tokyo, Japan) equipped with a manual injector model 7125 Rheodyne, a quaternary pump (Shimadzu LC-20AD, Tokyo, Japan), controlled by LC Solution software (Shimadzu, Tokyo, Japan) and equipped with a fluorescence detector Shimadzu RF-20A. Identification of UTs followed the protocols of the published methods [17,18]. The toxins were separated by a C8 column (Phenomenex, Luna 5 μm, 100A, 150 × 4.6 mm) using concentration gradient eluted with 50 mM ammonium formate pH 3.0 and methanol whose proportion increased from 35% to 70% along the run, at a flow rate of 0.7 mL/min. During the run, the fluorescence wavelengths varied as follows: *λ*exc = 280 nm and *λ*em = 383 nm to IS and IAA, *λ*exc = 265 nm and *λ*em = 290 nm to pCS.

### 4.4. Measurement of Plasma Levels of Inflammatory Biomarkers (IL-6, hsCRP, MCP-1, sICAM-1, sVCAM-1 and sFas) 

General markers of inflammation (IL-6, hsCRP), vascular inflammation chemokine (MCP-1), adhesion molecules (sICAM, sVCAM) and cell death marker (sFas) were analyzed.

The IL-6 concentration was performed by an enzyme-linked immunosorbent assay (ELISA), using commercially available kit (R&D Systems, Minneapolis, MN, USA). Ultra-high-sensitivity C-reactive protein (hsCRP) concentration was performed by the chemoluminescence technique (Immulite; DPC Biermann, Bad Nauheim, Germany). The concentration range to IL-6 and hsCRP were 0.5–1.500 pg/mL and 0.175–1.100 mg/L, respectively. 

Plasma concentration of MCP-1, soluble ICAM-1 (sICAM-1) and soluble VCAM-1 (sVCAM-1) were measured by ELISA using commercially available antibodies. The chemokines concentrations (pg/mL) and soluble adhesion molecules (ng/mL) were calculated against standard curves obtained using the corresponding recombinant molecules. The measuring range was 31.25–2.000 pg/mL for MCP-1 and 0.03–2 ng/mL for sICAM-1 and sVCAM-1. The intra-assay coefficients of variation (cv) for MCP-1, sICAM-1 and sVCAM-1 were 6.0%, 7.9% and 8.2%, respectively, and the inter-assay cv 6.2, 7.7% and 8.1%, respectively. Soluble Fas (sFas) was measured by ELISA using commercially available kit (OptEIA, PharMingen, San Diego, CA, USA) according to the manufacturer’s instructions. The lower limit of detection for sFas was 8 pg/mL. 

### 4.5. Biomarkers of the Uremic Cardiovascular Response (BUCVR)-sCD36, Fractalkine and sRAGE Plasma Levels

BUCVRs were measured by an enzyme-linked immunosorbent assay (ELISA) using commercially available kit (Cusabio Technology^®^-Houston, TX, USA for sCD36 and Quantikine^®^ Immunoassay-R&D Systems, Minneapolis, MN, USA for fractalkine and sRAGE). The ELISA system measuring range was 2.5–160 ng/mL for sCD36, 75–5000 pg/mL for sRAGE and 0.15–10 pg/mL for fractalkine. The intra-assay cv for sCD36, fractalkine, sRAGE were 7.5%, 7.2% and 8.2%, respectively, and the inter-assay cv 7.0%, 7.5% and 8.1% respectively.

### 4.6. Histology and Immunohistochemistry (IHC) of Arteries from CKD Patients and Healthy Controls 

Serial sections of external iliac and renal artery segments from stage 5 CKD patients and donor controls, respectively, were deparaffinized and rehydrated for routine IHC. Antigen retrieval was made by incubation with Immuno Retriver (Dako^TM^; Santa Clara, CA, USA), in a water bath for 30 min. To detect IL-6 (monoclonal mouse anti-IL-6 antibody [ABCAM-Cambridge, MA, USA]); MCP-1, CD36, fractalkine and fractalkine receptor (CX3CR1) (polyclonal rabbit anti-MCP-1, -CD36, -fractalkine and, -CX3CR1 antibodies [ABCAM-Cambridge, MA, USA]) sections were incubated in a humid chamber at 2–8 °C overnight. After incubation with a secondary antibody (Spring Reveal Complement; Spring Bioscience^TM^; Fremont, CA, USA), the sections were treated with an avidin-biotin-peroxidase complex (Spring Reveal Conjugate; Spring Bioscience^TM^; Fremont, CA, USA) and colored with diaminobenzidine (1:1; DAB chromogen-substrate solution-Dako^TM^; Santa Clara, CA, USA). The slides were counterstained with Harris hematoxylin and were mounted in histological resin for microscopy (Entellan, Merck^TM^, Kenilworth, NJ, USA). Positive and negative controls were included in all reactions. 

For the morphometric analysis, representative images of tissue expression of all biomarkers studied were captured with a Zeiss Axio Scan Slide Scanner (Carl Zeiss, Jena, Germany). Images were optimized in Adobe Photoshop CS6 V13.0 software, by removing the white areas. Twenty randomly selected medium-power fields (200× magnifications) in each slide were captured. The positive control MPF photomicrography was chosen as the “mask”, which contained adequate levels of positive tissue expression signal and was subsequently superimposed to the samples photomicrographs. Based on the ideal positive tissue immune expression signal obtained, the image analysis Image-Pro Plus 4.5^TM^ of Media Cybernetics^TM^ (Rockville, MD, USA) identified the positive areas and transform these results into positive tissue immune expression area per square micrometer (μm^2^). The area in μm^2^ obtained was divided by the total area of the MPF, thus generating a percentage value for each image. An average percentage of positive area was determined in 20 MPF images for each slide.

### 4.7. Non-Invasive Evaluation of Atherosclerotic CVD

Carotid ultrasonographic examination was performed in the afternoon of the day of blood collection, to avoid interference from daily fluctuations of inflammation biomarkers. The right and the left carotid arteries were examined with a high-resolution duplex scanner (Apogee Plus; ATL-Phillips, Washington, DC, USA) using a 5- to 10-MHz linear array transducer. The subjects were examined in dorsal decubitus with the head slightly turned to the sonographer. All the evaluations were performed by the same trained sonographer. The distal wall of the common carotid arteries (CCA), 0.5–1.0 cm proximal to the beginning of the carotid bulb, was used to determine the intima-media thickness (IMT) on each side at 0.5-cm intervals. The IMT was defined as the distance between the leading edge of the lumen-intima echo and the leading edge of the media-adventitia echo. Measurements were always performed on arterial segments without plaque. The intima-media layer was analyzed for the presence of plaque formation and CVD history. Plaque was defined as a clearly identified area of focal increased thickness (>1 mm) from the intima-media layer.

### 4.8. Cardiovascular Events during the Follow Up

For the analysis of predictors of cardiovadcular events during the follow up, total cardiovascular mortality, non-fatal MI, revascularization, amputation or non-fatal stroke. This information was obtained by a detailed analysis of medical records and patient interview (including telephone contacts for confirmation). Patients who lost to follow up were censored. 

### 4.9. Statistical Analysis

Results were expressed as the mean ± SD, median and range or frequency. The Kolmogorov-Smirnov test was used to assess normality of the quantitative variables. Variables that did not meet this condition underwent a logarithmic transformation. Student *t*-test for independent samples was used for comparison between two groups in relation to quantitative variables. Pearson’s coefficients (*r*) was used for estimating correlation between quantitative variables. To examine the associations of eGFR, UT plasma levels, inflammatory biomarkers and BUCVR with all causes mortality risk after 5.2 years of follow-up and adjustment for age, sex, diabetes and CVD, univariate analyses were performed using the Cox regression model and the Wald test, for assessing the significance of co-variables. *p* values < 0.05 were considered statistically significant. Data were analyzed with the computer program IBM SPSS v.20 (SPSS Inc., Chicago, IL, USA) and STATA 14^®^ (StataCorp, College Station, TX, USA).

## Figures and Tables

**Table 1:** Main clinical and laboratory characteristics of the study population (cohort 1).

Analyzed Parameters					
Patients, *n*	67				
Traditional risk factors	CKD 1 (*n* = 7)	CKD 2 (*n* = 15)	CKD 3 (*n* = 20)	CKD 4 (*n* = 16)	CKD 5 (*n* = 9)
Mean age ± SD, years	45 ± 16.7	54 ± 11.2	61 ± 11.2	56 ± 14.9	61 ± 8.7
Gender, % females	6	12	21	10	8
Race, % caucasians	9	17	28	22	14
Mean BMI ± SD	29.6 ± 5.3	28.2 ± 3.7	28.4 ± 4.0	28.4 ± 4.5	28 ± 6.1
Diabetes, %	3	9	12	9	3
Hypertension, %	3	12	19	15	8
CVD, %	9	13	15	10	6
Dyslipidemia, %	5	6	6	5	5
Smoking, %	2	3	3	2	3
LVH, %	-	6	6	9	3
Plaques, %	3	5	8	9	6
**Primary Kidney Disease**					
Diabetic nephropathy, %	3	9	9	5	5
Glomeruloesclerosis hypertensive, %	-	5	10	9	5
Chronic glomerulonephritis, %	6	2	2	5	-
Others and unknown, %	2	3	3	3	2
**Laboratory Parameters**					
GFR (CKD-EPI), mL/min Median (range)	101.0 90.6–125	74.6 60.1–89	41.4 30–59.3	19.4 15–27.5	12.3 8.1–14.4
Proteinuria, mg/24 h	222.1	277.0	1091.0	879.0	3232.0
Albumin, mg/dL	4.2	4.0	3.8	3.8	3.8
Glucose, mg/dL	85.5	92.2	106.4	122.8	107.8
HDL cholesterol, mg/dL	47.6	41.0	44.4	38.2	39.7
LDL cholesterol, mg/dL	112.0	100	122	109.6	135.4
Triglycerides, mg/dL	175.1	217.2	168.2	202.3	191.1
Calcium, mg/dL	9.2	9.3	8.9	9.0	9.2
Phosphorus, mg/dL	3.3	3.7	3.7	4.3	5.0
Hemoglobin, g/dL	14.6	14.4	13.9	12.0	12.3

CVD = Cardiovascular disease, LVH = Left ventricular hypertrophy, BMI = Body mass index; GFR (CKD-EPI) = Glomerular filtration rate estimated by Chronic Kidney Disease Epidemiology Collaboration equation, LDL = Low-density lipoprotein; HDL = High-density lipoprotein.

**Table 2:** Main clinical and laboratory characteristics chronic kidney disease (CKD) transplant recipients (cohort 2).

Analyzed Parameters		
Traditional risk factors	Control group (*n* = 12)	CKD group (*n* = 14)
Mean age ± SD, years	41.6 ± 10.1	37.1 ± 13.1
Gender, % females	50	43
Mean BMI ± SD	24.7 ± 3.5	24.3 ± 3.2
Hypertension, %	0	100
**Primary Kidney Disease**		
Hypertensive nephrosclerosis, %	-	43
Chronic glomerulonephritis, %	-	43
Others and unknown, %	-	14
**Laboratory Parameters**		
Creatinine, mg/dL	0.8 ± 0.2	8.6 ± 3.2
Glucose, mg/dL	93 ± 17.2	112.0 ± 28.1
HDL cholesterol, mg/dL	50.1 ± 10.7	45.4 ± 17.4
LDL cholesterol, mg/dL	112 ± 28.2	114 ± 30.3
Calcium, mg/dL	9.1 ± 0.5	9.5 ± 0.9
Phosphorus, mg/dL	4.01 ± 1.0	5.8 ± 1.9
Hemoglobin, g/dL	13.5 ± 1.9	10.6 ± 1.5

BMI = Body mass index; LDL = Low-density lipoprotein; HDL = High-density lipoprotein.

**Table 3:** Uremic toxins, Inflammatory biomarkers and BUCVR of the study population.

Analyzed Parameters	Mean ± SD	Median (Range)
Uremic Toxins		
IS, uM	15.8 ±19.4	7.6 (1.1–100.5)
pCS, uM	91.5 ± 92.2	60.7 (0.9–501.0)
IAA, uM	0.80 ± 0.57	0.66 (0.06–3.28)
**Inflammatory Biomarkers**		
IL-6, pg/mL	4.91 ± 3.37	3.69 (0.67–11.0)
hsCRP, mg/L	5.18 ± 6.66	2.8 (0.30–39.9)
MCP-1, pg/mL	105.9 ± 31	102.9 (54.4 0–229.0)
sVCAM, ng/mL	806 ± 392	689 (378–1849)
sICAM, ng/mL	81 ± 14.5	80.8 (39.6–156.8)
sFas pg/mL	1339 ± 659	1253 (306–4181)
**BUCVR**		
sCD36, ng/mL	66.6 ± 34.4	60.9 (5.8–157.9)
sRAGE, pg/mL	2594 ± 1115	2552 (795–4827)
Fractalkine, ng/mL	1.30 ± 0.46	1.24 (0.27–2.42)

IS (indoxyl sulfate), pCS (para-cresyl sulfate), IAA (indole-3-acetic acid); IL-6 (Interleukin-6), hsCRP (High sensitivity C-reactive protein), MCP-1 (Monocyte chemoattractant protein-1), sVCAM-1 (soluble vascular adhesion molecule-1), sICAM-1 (soluble intercellular adhesion molecule-1); BUCVR = biomarkers of the uremic cardiovascular response, sRAGE (soluble receptor for advanced glycation end products).

**Table 4:** Correlations between uremic toxins and inflammatory biomarkers.

	IS		pCS		IAA	
	*r*	*p*	*r*	*p*	*r*	*p*
IL-6, pg/mL	0.13	0.29	0.236	0.055	0.078	0.53
hsCRP, mg/L	0.10	0.42	0.024	0.84	0.020	0.87
MCP-1, pg/mL	0.38	0.001	0.42	<0.001	0.29	0.015
sVCAM, ng/mL	0.29	0.019	0.29	0.017	0.27	0.025
sICAM, ng/mL	0.047	0.71	0.12	0.34	0.087	0.48
sFas, pg/mL	0.41	0.001	0.46	<0.001	0.19	0.13

IL-6 (Interleukin-6), hsCRP (High sensitivity C-reactive protein), MCP-1 (Monocyte chemoattractant protein-1), sVCAM-1 (soluble vascular adhesion molecule-1), sICAM-1 (soluble intercellular adhesion molecule-1), IS (indoxyl sulfate), pCS (para-cresyl sulfate), IAA (indole-3-acetic acid).

**Table 5:** Correlations between uremic toxins and BUCVR.

	IS		pCS		IAA	
	*r*	*p*	*r*	*p*	*r*	*p*
sCD36, ng/mL	0.62	<0.001	0.55	<0.001	0.52	<0.001
sRAGE, pg/mL	0.48	<0.001	0.48	<0.001	0.20	0.108
Fractalkine, ng/mL	0.77	<0.001	0.77	<0.001	0.41	<0.001

BUCVR (biomarkers of the uremic cardiovascular response), UTs (uremic toxin), IS (indoxyl sulfate), pCS (para-cresyl sulfate), IAA (indole-3-acetic acid), sRAGE (soluble receptor for advanced glycation end products).

**Table 6:** Correlations between inflammatory biomarkers (IL-6, hsCRP, MCP-1, sVCAM-1, sICAM-1, sFas) and BUCVR (sCD36, sRAGE, Fractalkine).

	MCP-1		sICAM		sCD36		sRAGE		Fractalkine	
	*r*	*p*	*r*	*p*	*r*	*p*	*r*	*p*	*r*	*p*
IL-6, pg/mL	0.36	0.003	NS		NS		NS		NS	
hsCRP, mg/L	NS		0.37	0.002	NS		NS		NS	
MCP-1, pg/mL	-		NS		0.30	0.015	0.28	0.022	0.38	0.002
sVCAM, ng/mL	0.41	0.001	NS		0.33	0.006	NS		NS	
sICAM, ng/mL	NS		-		NS		NS		NS	
sFas, pg/mL	NS		NS		0.47	<0.001	0.39	0.001	0.43	<0.001
sCD36, ng/mL	0.30	0.015	NS		-		0.42	<0.001	0.73	<0.001
sRAGE, pg/mL	0.28	0.022	NS		0.42	<0.001	-		0.50	<0.001
Fractalkine, ng/mL	0.38	0.002	NS		0.73	<0.001	0.50	<0.001	-	

IL-6 (Interleukin -6), hsCRP (High sensitivity C-reactive protein), MCP-1 (Monocyte chemoattractant protein-1), sVCAM-1 (soluble vascular adhesion molecule-1), sICAM-1 (soluble intercellular adhesion molecule-1), BUCVR (biomarkers of the uremic cardiovascular response). NS (Not significant).

**Table 7:** Comparing cases with and without plaques with respect to uremic toxins, inflammatory biomarkers and BUCVR.

		PLAQUES		
		No (*n* = 44)	Yes (*n* = 20)	*p*
UT	IS, uM	16.6 ± 21.3	14.9 ± 16.0	0.957
	pCS, uM	74.9 ± 66.1	113 ± 96.5	0.160
	IAA, uM	0.75 ± 0.47	0.92 ± 0.77	0.378
Inflammatory Biomarkers	IL-6, pg/mL	4.4 ± 3.1	5.9 ± 3.6	0.106
	hsCRP, mg/L	5.1 ± 7.5	5.3 ± 4.6	0.921
	MCP-1, pg/mL	102.9 ± 27.9	113.4 ± 36.3	0.212
	sVCAM-1, ng/mL	772.7 ± 341.1	884.1 ± 480.4	0.293
	sICAM-1, ng/mL	81.0 ± 9.5	82.2 ± 22.3	0.762
	sFas, pg/mL	1418.8 ± 694.8	1224.0 ± 584.7	0.289
BUCVR	sCD 36, ng/mL	61.0 ± 30.2	79.1 ± 40.1	0.049
	sRAGE, pg/mL	2661 ± 1108	2508 ± 1129	0.611
	Fractalkine, ng/mL	1.25 ± 0.45	1.40 ± 0.45	0.223

UT (uremic toxin), IS (indoxyl sulfate), pCS (para-cresyl sulfate), IAA (indole-3-acetic acid); IL-6 (Interleukin-6), hsCRP (High sensitivity C-reactive protein), MCP-1 (Monocyte chemoattractant protein-1), sVCAM-1 (soluble vascular adhesion molecule-1), sICAM-1 (soluble intercellular adhesion molecule-1); sRAGE (soluble form receptor for advanced glycation end products), BUCVR (biomarkers of the uremic cardiovascular response); Mean ± SD; Test *t* Student (*p* < 0.05).
